# Deletion of the miR‐25/93/106b cluster induces glomerular deposition of immune complexes and renal fibrosis in mice

**DOI:** 10.1111/jcmm.16721

**Published:** 2021-07-01

**Authors:** Hongchuang Ma, Xiang Li, Shanshan Yu, Yanling Hu, Meixiang Yin, Fubin Zhu, Licheng Xu, Tianhe Wang, Huiyan Wang, Hongzhi Li, Binghai Zhao, Yadong Huang

**Affiliations:** ^1^ Department of Cell Biology Jinan University Guangzhou China; ^2^ Jilin Collaborative Innovation Center for Antibody Engineering Jilin Medical University Jilin China; ^3^ Nephrosis Precision Medicine Innovation Center University of Beihua School of Medicine Beihua University Jilin China; ^4^ Shenzhen Samii Medical Center Guangdong China

**Keywords:** fibrosis, IgA nephropathy, immune complex, microRNA

## Abstract

IgA nephropathy (IgAN), the most common form of primary glomerulonephritis, is caused by immune system dysfunction and affects only the kidneys. miRNA was involved in IgAN, in which their roles are still unknown. Herein, we found increased glomerular medulla size, proteinuria, kidney artery resistance, kidney fibrosis and immune complex deposition in 5‐month miR‐25/93/106b cluster knockout (miR‐TKO) mice. In vitro, the inhibition of miR‐25 cluster could promote cell proliferation and increase fibrosis‐related protein and transferrin receptor (TFRC) expression in human renal glomerular mesangial cell (HRMC). Luciferase assay revealed that inhibition of miR‐93/106b cluster could upregulate *Ccnd1* expression through direct binding with the 3’UTR of *Ccnd1*. Conversely, inhibition of *Ccnd1* expression prevented miR‐93/106b‐induced effect in HRMC. These findings suggested that miR‐25 cluster played an important role in the progression of IgAN, which provided new insights into the pathogenesis and treatment of IgAN.

## 1 INTRODUCTION

IgAN is one of the most common primary glomerulonephritis,^1^ about 15%‐40% IgAN eventually progresses into end‐stage renal disease (ESRD); therefore, early diagnosis and treatment are essential to prevent or delay the progression of IgAN.^2^, ^3^, ^4^, ^5^ So far, the diagnosis of IgAN still requires histologic diagnosis, mainly featured as abnormal IgA and complement C3 deposition or IgA co‐deposition with or without IgM/G in the glomerular mesangium.^6^, ^7^ To date, the pathogenesis of IgAN is generally believed to be the four‐strike theory: increased abnormal IgA(hit 1), the production of IgA autoantibodies(hit 2) and the antibody recognizes antigen to form an immune complex(hit 3), which is deposited in the mesangial region of the glomerulus and causes proliferation of mesangial cells(hit 4).^8^ Although many researches have been made on the basis and clinical aspects of IgA nephropathy, the exact pathogenesis of IgA nephropathy has not been clarified.

MicroRNA is a single‐stranded RNA molecule about 21 to 23 nucleic acids in length. It is mainly involved in the regulation of gene expression at the post‐transcriptional level and played an important role in many physiological and pathological processes.^9^, ^10^, ^11^ MicroRNA is involved in every process of cells, and regulatory obstacles of microRNA are involved in a variety of human diseases including chronic kidney disease(CKD).^12^, ^13^

Many miRNAs were involved in IgAN, such as let‐7b,^14^ miR‐148b,^15^ miR‐133a/b,^16^ miR‐223^17^ and miR‐106b.^16^ Previously, we showed inhibition of miR‐25 by commercial miRNA antagomir, which caused high blood pressure and kidney dysfunction.^18^ It hinted us that miR‐25 may be involved in CKD, but belongs to which type of CKD and the exact molecular mechanism under such CKD was unclear. Simultaneously, delivery of LNA‐modified anti‐miRNA oligonucleotides in vivo has also been reported to potentially cause off‐target and short‐term effects compared with miRNA‐null mice.^19^ Furthermore, many miRNAs located in same cluster, cotranscribed and targeted same seed sequences. These miRNAs always express functional redundancy, which is likely that deletion of single miRNA could have been made up for other increased miRNA existed in same cluster.^20^ Herein, we employed miR‐25/93/106b cluster knocking‐out (miR‐TKO) mice and showed that 5‐month‐old miR‐TKO mice induce deposition of glomerular mesangial immune complexes, increased glomerular fibrosis and renal arterial resistance, and activated renin‐angiotensin‐aldosterone system. Inhibition of miR‐25 cluster promoted cell proliferation and fibrosis in human renal glomerular mesangial cell (HRMC) by targeting *Ccnd1*. These findings will provide new insights into the pathogenesis and therapeutic target of IgAN.

## 2 MATERIALS AND METHODS

### 2.1 Animal treatment

The miR‐25 cluster knockout mice (#008460) were obtained from JAX laboratory. The miR‐25 cluster genotypes were confirmed by PCR using primers including common: TCCACTGCTCTGGTGAGTGG, wild type: AGGAAGTACCCACAGTGCGG and mutant: TGCTCCAGCTTCAAGCCTGG. All mice were kept in specific pathogen‐free conditions, and Beihua University Animal Care and Veterinary Services approved all protocols. The investigations conform to the Guide for the Care and Use of Laboratory Animals published by the National Institutes of Health. We used same‐sex and 5‐month‐old male miR‐TKO mice (littermates) in this study unless stated otherwise. Echocardiography (VEVO 2100, STTARR) or MRI (BioSpec, Bruker, Ettlingen, Germany) was performed on 5‐month‐old male mice before killing the animals. Kidneys and serum were collected, and the majority of the tissues were stored at −80℃.

### 2.2 Cell culture

The human renal mesangial cell (HRMC) obtained from ScienCell Research Laboratories is isolated from human renal tissue. It was incubated with an atmosphere of 5% CO_2_/95% air and used with mesangial cell growth supplement (MsCGS, ScienCell Research Laboratories, Cat#4252) and 10 mL of foetal bovine serum. HRMCs were transfected with miRNA‐25/93/106b inhibitor (100 nmol/L; Rabio Co. Guangzhou, China) using Lipofectamine 2000 (Invitrogen Life Technologies, Carlsbad, CA, USA) according to the manual. For miRNA‐25/93/106b mimic transfection, we transfected miRNA‐25/93/106b mimic (50 nmol/L; Rabio Co. Guangzhou, China) into HRMC cells. Scrambled controls were used in parallel. Ccnd1 siRNA (100 nmol/L; Rabio Co. Guangzhou, China) was transfected into HRMCs, and scrambled siRNA controls were used in parallel.

### 2.3 Luciferase reporter assay

*Ccnd1* gene 3’‐UTR luciferase vector containing the miR‐93/106b response elements was amplified by PCR from mouse cDNA. Plasmid DNA and miR‐25/93/106b inhibitor (100 nmol/L) and mimic (50 nmol/L) were co‐transfected into HEK 293A cells for 48 hours. Luciferase activity was measured using a SpectraMax M5 (Molecular Devices, Sunnyvale, CA, USA) and normalized by measuring β‐galactosidase activity. The primers used to generate specific fragments for the mouse *Ccnd1* gene 3'‐UTR are listed in Table S1.

### 2.4 Histological analyses of the kidney samples

For immunofluorescence and immunochemistry, mouse monoclonal antibody against α‐SMA, IgG and IgA, C3, TFRC, IgM or FN (Abcam) and goat polyclonal anti‐mouse secondary antibody and rabbit HRP (Abcam) were used. For the quantitative morphometry, cells stained in 10 randomly selected micrographs were counted using Image ProPlus software (Image‐Pro Plus, Media Cybernetics). For PAS staining, kidney paraffin sections (5 μm) were stained using PAS (ScyTek Laboratories Inc Logan) kits according to the manufacturer's protocols.

### 2.5 Western blotting and ELISA assay

The kidney tissues were homogenized in lysis buffer (Thermo Fisher Scientific) containing 0.025 M Tris, 0.15 M NaCl, 0.001 M EDTA, 1% NP‐40, 5% glycerol, pH 7.4, protease inhibitor (Roche Hong Kong Limited, Hong Kong, China). Total proteins were quantified using the BCA assay kit (Thermo Scientific). Samples (20 µg/lane) were resolved by SDS‐PAGE, blotted and probed with the following primary antibodies: the β‐actin (Abcam Hong Kong Ltd.) blot was used as a striped membrane. An ELISA for IgA (GeneTex), IgG and IgM (Abcam) was performed according to the manufacturer's instructions using a commercially available kit for mouse. An ELISA for renin, angiotensin 1/2, was performed using a commercially available kit for mouse (Santa Cruz) according to the manufacturer's instructions.

### 2.6 Ultrastructural analysis

Kidney tissues were fixed in 2.5% glutaraldehyde in 0.1 M phosphate buffer (pH 7.4) at 4℃ for 24 hours. The samples were then washed with phosphate buffer (0.1 M, pH 7.4) for 12 hours and post‐fixed for 20 minutes in 1% OsO4 in 0.1 M phosphate buffer (pH 7.4). The samples were then washed with phosphate buffer (0.1 M, pH 7.4) for 30 minutes, dehydrated and embedded in Epon. Thin sections (50 nm) were placed on copper grids and stained for 30 minutes with a 2% uranyl acetate solution and a 1% solution of lead citrate. A JEM‐1010 transmission electron microscope was used to visualize the ultrastructure. Ten randomly selected areas from each specimen were photographed and analysed using Image ProPlus software (Image‐Pro Plus, Media Cybernetics).

### 2.7 Magnetic resonance imaging

The animals were anaesthetized by inhalation of 2% isoflurane and a mixture of O2 and N2O. Bed temperature was maintained at 37.5℃ by applying warm water circulation. All MRI data were collected at 9.4 T (Bruker Biospec 94/20 USR; Bruker Biospin, Ettlingen, Germany). Mice were placed in the prone position. Then, a 1 H volume coil (Bruker Biospin) was used for both RF transmission and signal reception and tuned to 1H resonance frequencies (400.31 MHz). Scout images were acquired using a gradient echo sequence with the following imaging parameters: field of view (FOV), 40 × 40 mm^2^; image size, 256 × 256; repetition time (TR)/echo time (TE), 4/1.5 ms; flip angle (FA), 8°; number of slices, 3 (axial), 8 (coronal) and 3 (sagittal); slice thickness (TH), 1 mm; and 6 signal averages. For T1 imaging, IG_FLASH was used and the parameters were as follows: echo time, 3.0 ms; repetition time, 202.235 ms; flip angle, 40.0°; oversampling, 15; image size, 320 × 320; FOV, 40 × 40 mm2; 15 slices were acquired (coronal). For T2 imaging, TurboRARE was used and the parameters were as follows: echo time, 30.0 ms; repetition time, 1800 ms; averages, 9; echo spacing, 10.0 ms;rare factor, 8; image size, 256 × 256; FOV, 40 × 40 mm; 15 (coronal) and 20 (axial) slices were acquired, respectively.

### 2.8 RNA extraction and analysis

For the tissue extraction, RNAs were extracted from the kidneys using a miRNA isolation kit (Ambion Inc) to separate into large and small RNAs according to the manufacturer's instructions. MicroRNA real‐time PCR was used with a final reaction volume of 20 μL containing 9 μL Fast Start Universal SYBR Green Master Mix (Roche), 7.4 μL nuclease‐free water, 0.8 μL miRNA primers (Rabio Co. Guangzhou, China) and a 2 μL RT product. The data were normalized to RNU6B small nuclear RNA by a standard curve method to account for differences in reverse transcription efficiencies and the amount of template in the reaction mixtures. For the mRNA expression analysis, first‐strand cDNA was synthesized from 1 μg of total RNA using Moloney murine leukaemia virus reverse transcriptase and a Random Primers kit (Promega Corp.). The ribosomal protein S16 mRNA level served as the internal control. The primer sequences used are listed in Table S2‐S3.

### 2.9 RNA sequencing

All the large RNAs were isolated using the mirPremier™ microRNA Isolation Kit (Ambion Inc). The mRNA libraries were constructed using the TruSeq Stranded Total RNA Library Prep Kit (NEB Inc, Ipswich). Sequencing was conducted using Hi‐Seq 2000 sequencers with PE50 at BGI (Shenzhen, China). The RNA‐seq data from miR‐25 cluster knock out mice kidney are at BioProject under accession numbers PRJNA527149.

### 2.10 RNA‐seq data analysis

The raw RNA‐Seq data were filtered by trimmomatic (v0.33).^21^ The clean reads were mapped using Tophat2 (v2.1.1) and then counted into different gene regions as the corresponding GTF files (mm9, GRCh38 or hg19, GRCh37 genome assembly) by featureCount (v1.5.3).^22^ The counts of mapped genes are normalized by DEGseq (v1.32.0) with default parameters.^23^ The significant genes are selected as suggested value *P* < .001 and abs(log2foldchange) >0.5 or the program default significant criteria. Target genes of the miRNAs were determined using the Bioconductor Package‐targetscan. Mm.eg.db and targetscan. Hs.eg.db.^24^

## 3 RESULTS

### 3.1 Deletion of miR‐25 cluster induced proteinuria and kidney medullary thicken

**FIGURE 1:**
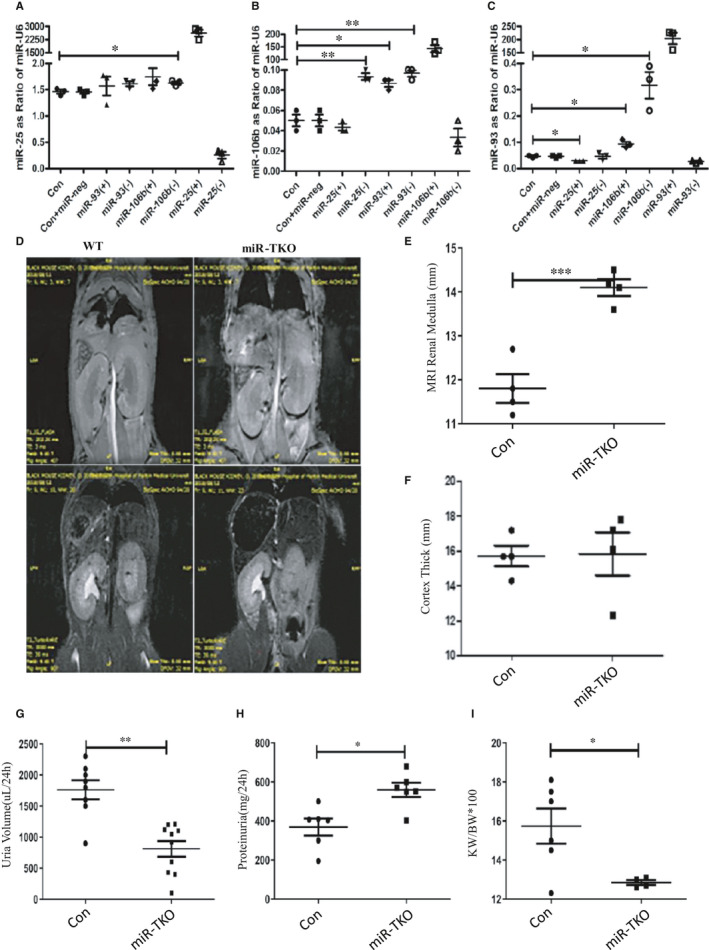
Deletion of miR‐25 cluster induced proteinuria and kidney medullary thicken. A‐C, Quantification of miR‐25, 106b and 93 expression in cells treated by miR‐25/93/106b mimic or inhibitor; D, representative image MRI T1/2 (second and third panel; WT, n = 10; KO, n = 10) from 5‐mo‐old male wild‐type and miR‐TKO mice; E and F, quantification of MRI T2 medulla and cortex distance from wild‐type and miR‐TKO mice; G and H, quantification of 24‐h urine volume (n = 8) and proteinuria (n = 6) from wild‐type and miR‐TKO mice; I, quantification of ratio of kidney weight to bodyweight from wild‐type (n = 6) and miR‐TKO mice (n = 6); *** and ***indicate *P* < .05, *P* < .01 and *P* < .001, respectively. Data are shown as the mean ± SEM

Firstly, we measured the expression levels of other miRNAs in miR‐25/93/106b cluster when the expression level of a single miRNA in this cluster was inhibited. As expectedly, the expression level of miR‐106b was increased significantly when the expression level of miR‐25 and miR‐93 was inhibited, and the expression level of miR‐93 was also upregulated when the expression level of miR‐106b was inhibited (Figure 1A‐C). Thus, this suggested that the miR‐25/93/106b cluster might have complementarity of miRNA functions. To characterize the role of miR‐25/93/106b cluster in kidney disease, we employed miR‐25/93/106b cluster knocking‐out mice from JAX laboratory. To detect the kidney pathologic phenotype in miR‐TKO mice, a non‐invasive magnetic resonance imaging (MRI) was used. Through high‐sensitivity MRI, we found medullary was indeed thickened and cortico‐medullary boundary was obscured in miR‐TKO mice as compared to the wild‐type mice (Figure 1D, E). Although the medullary structure changed noticeably, the cortex thickness was not significantly different (Figure 1F). Such kidney structure alteration reminded us measuring the urine feature, which showed decreased 24‐hour urine volume in miR‐TKO mice (Figure 1G), and it accompanied with increased proteinuria in miR‐TKO mice compared with WT mice (Figure 1H). We also found the ratio of kidney weight to bodyweight decreased in miR‐TKO mice compared with WT mice (Figure 1I).

### 3.2 Deficiency of miR‐25 cluster induces immune complex deposition in glomerular mesangial

**FIGURE 2:**
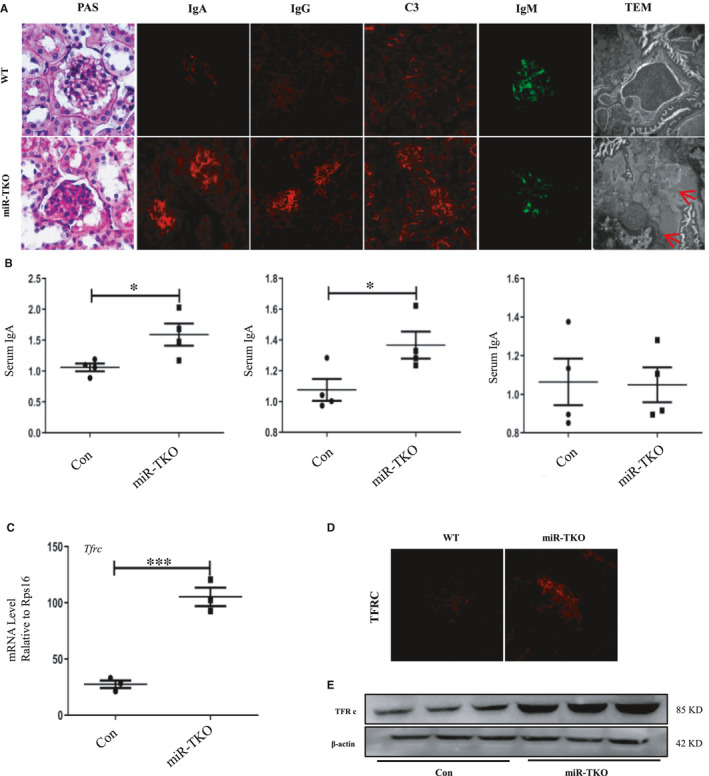
Deficiency of miR‐25 cluster induces immune complex deposition in glomerular mesangial. A, Representative image of PAS, IgA, IgG, C3, IgM (WT and KO, n = 8), TEM (WT, n = 10; KO, n = 20) staining from kidney sections of 5‐mo‐old miR‐TKO and wild‐type mice. The bars in immunofluorescence indicate 10 μm, and bars in TEM indicate 2 μm;B, Quantification of serum IgA, IgG and IgM from the wild‐type(n = 4) and 5‐mo‐old (n = 4) miR‐TKO mice; C, quantification of Tfrc mRNA expression from the wild‐type(n = 3) and 5‐mo‐old (n = 3) miR‐TKO mice D and E, Represented image of TFRC immunofluorescence (n = 5) and Western blotting (n = 3)from the wild‐type and 5‐mo‐old (n = 3) miR‐TKO mice; ** and *** indicate *P* < .01 and *P* < .001, respectively. Data are shown as the mean ±SEM

In order to understand the basis for these structural and functional abnormalities, we performed standard histomorphometry, immunofluorescence and electron microscopy on the kidneys from miR‐TKO and WT mice. The renal PAS staining of 5‐month‐old miR‐TKO mice displayed mild segmental multifocal expansion of the mesangial matrix and tubular atrophy (Figure 2A [first panel]). Immunofluorescence demonstrated IgA, IgG and C3 deposition in the glomerular mesangium of miR‐TKO mice which was absent in age‐matched WT mice (Figure 2A [second to fifth panel]). Immune complex deposits were confirmed by electron microscopy (Figure 2A [sixth panel]). miR‐TKO kidneys also had weak glomerular deposition of IgM; however, there is no significant difference from that observed in WT mice (Figure 2A [fifth panel]). We furtherly measured the serum IgA and IgG levels which increased significantly in miR‐TKO mice compared with WT mice; however, serum IgM has no obvious alteration(Figure 2B). TFRC is IgA receptor in glomerular mesangial cells and was activated in IgAN. Real‐time PCR and immunofluorescence or Western blotting detection showed upregulated TFRC expression levels in the mesangial region of the glomerulus (Figure 2C‐E).

### 3.3 Deficiency of miR‐25 cluster activates the Renin‐Angiotensin‐Aldosterone System

**FIGURE 3:**
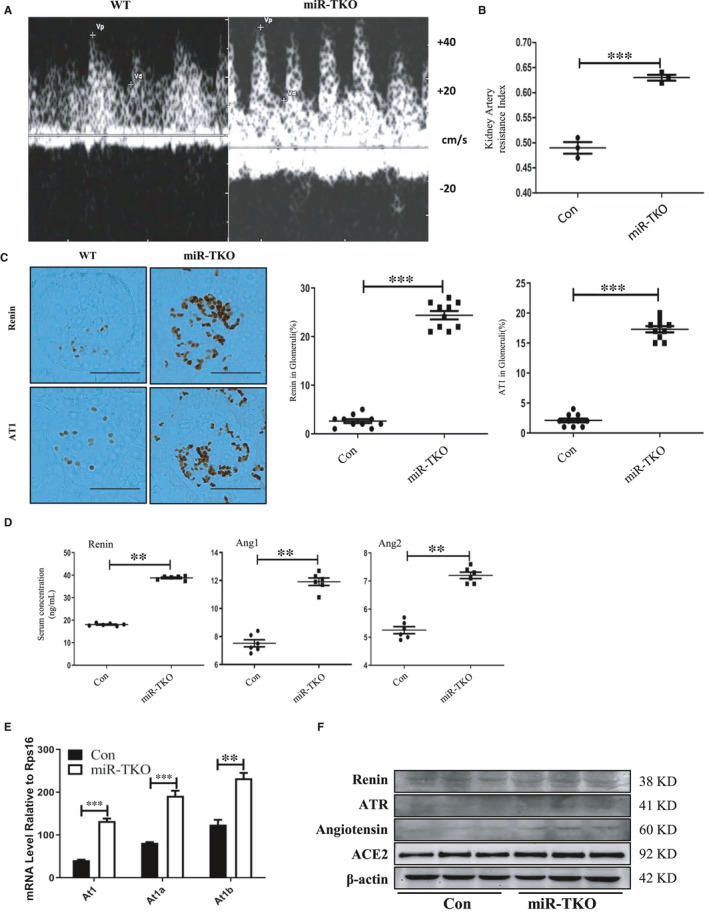
Deficiency of miR‐25 cluster associated with Increased KRI and Activated Renin‐Angiotensin‐Aldosterone System. A, Represented image of B‐mode ultrasonography of the kidney from miR‐TKO and wild‐type mice; B, quantification of kidney artery resistance index (KRI) in 5‐mo‐old male WT and miR‐TKO mice; C, representative images of renin and AT1 from 5‐mo‐old miR‐TKO and wild‐type mice, right panel is quantification of the renin and AT1‐positive area within the glomeruli; D, quantification of serum renin‐angiotensin 1/2‐aldosterone‐related protein in 5‐mo‐old miR‐TKO and wild‐type mice; E, Quantitative real‐time PCR analysis for *At1*, *At1a* and At1b from 5‐mo‐old miR‐TKO and wild‐type mice; F, Western blot analyses for renin, ATR, angiotensin and ACE2 from 5‐mo‐old miR‐TKO and wild‐type mice. *, ** and *** indicate *P* < .05, *P* < .01 and *P* < .001, respectively. Data are shown as the mean ± SEM

Hypertension, as target for treatment in clinic, always accompanied with IgAN.^25^ Renal arterial resistance index (KRI, peak systolic and end‐diastolic flow velocity/peak systolic flow velocity) is measured by colour Doppler and can reflect not only the onset and progression of renal disease, but also hypertension.^26^ According to the ultrasound, it showed significantly increased KRI in miR‐TKO mice compared with wild‐type mice (Figure 3A, B). This strongly reminded us that deficiency of miR‐25 cluster maybe resulted in hypertension. Abnormal RAS system was related to hypertension; here, we showed increased renin and AT1 expression in miR‐TKO mice compared with WT mice (Figure 3C). This variation was as same as upregulation of the RAS‐related protein (renin, angiotensin 1, angiotensin 2, aldosterone) which was detected in the serum of miR‐TKO mice as demonstrated by ELISA (Figure 3D). We detected significantly elevated mRNA expression level of *At1a*, *At1b* and *At1* in kidney of miR‐TKO mice by qPCR compared with wild‐type mice (Figure 3E). MiR‐TKO mice also existed increased renin, ATR (angiotensin 2 type 1 receptor), angiotensin and ACE2 (angiotensin‐converting enzyme 2) protein expression level by WB measurement (Figure 3F).

### 3.4 Deletion of miR‐25 cluster induces kidney fibrosis

**FIGURE 4:**
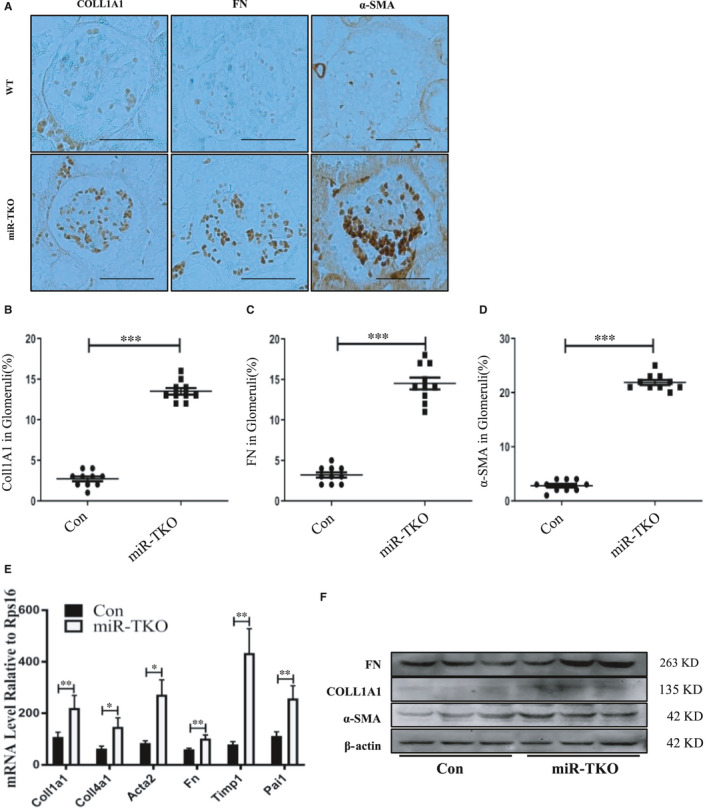
Deletion of miR‐25 cluster induces kidney fibrosis. A, Representative image of COLL1A1, FN and α‐SMA staining (5‐mo‐old) from kidney sections of miR‐TKO and wild‐type mice; B‐D, Quantification of the Collagen 1a1, FN and α‐SMA‐positive area within the glomeruli; E and F, t‐e mRNA and protein expression level of fibrosis relative gene from kidney tissue of miR‐TKO and wild‐type mice. *, ** and *** indicate *P* < .05, *P* < .01 and *P* < .001, respectively. Data are shown as the mean ± SEM

Although IgAN pathogenesis is not originated from kidney, it only affects kidney. Kidney fibrosis is one feature of IgAN; it represented acute clinical symptom and suggests progression to end‐stage renal disease. Can the deletion of miR‐25/93/106b cluster cause renal fibrosis? We observed miR‐TKO mice had increased deposition of fibrillar collagens, mainly in the glomeruli, compared with the wild‐type mice as revealed by FN (fibronectin), a‐SMA (alpha smooth muscle actin) and COLL1A1 (collagen 1) staining of the kidneys (Figure 4A‐D). By employing qPCR, we also analysed fibrosis‐related genes and found increased mRNA expression levels of *collagen 1a* (I), *collagen 4a* (IV), *Fn* (*Fibronectin), Acta2, Timp1 (tissue inhibitor of metalloproteinases 1) and Pai1 (plasminogen activator inhibitor type 1)* in 5‐month‐old miR‐TKO mice kidneys (Figure 4E). Consistent with previous results, we detected upregulated protein level of COLL1A1, FN, a‐SMA in 5‐month‐old miR‐TKO kidney by employing WB, compared with WT mice (Figure 4F).

### 3.5 Inhibition of miR‐25 cluster promotes HRMC proliferation and fibrosis

**FIGURE 5:**
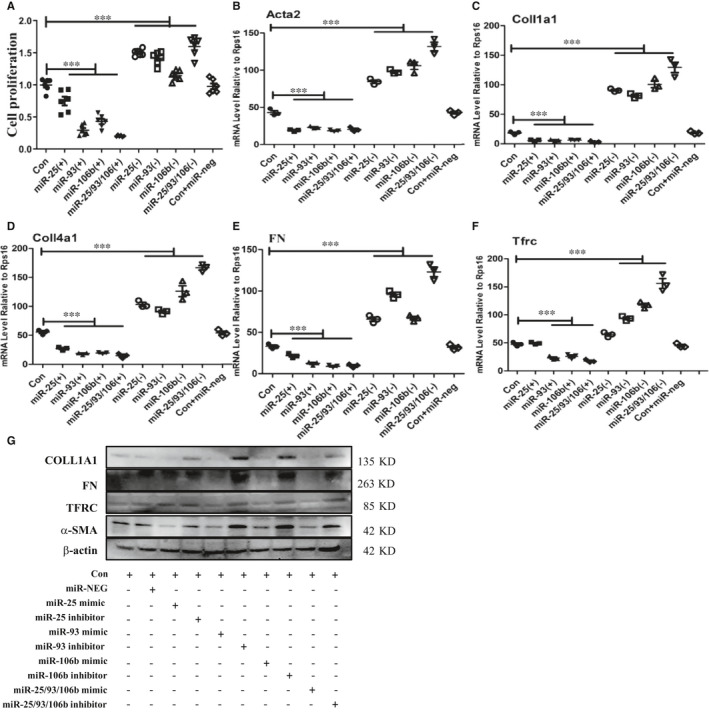
Inhibition of miR‐25 cluster promotes HRMC proliferation and fibrosis. A, Cell proliferation level analysis in HRMC treated by miR‐25 mimic or inhibitor, miR‐93 mimic or inhibitor, and miR‐106b mimic or inhibitor; B‐F, quantitative real‐time PCR analysis of *Coll1a1*, *Coll4a1*, *Fn*, *Acta2* and *Tfrc* level in HRMC treated by miR‐25 mimic or inhibitor, miR‐93 mimic or inhibitor, and miR‐106b mimic or inhibitor; G, Western blot analysis for TFRC, COLLA1, FN and α‐SMA proteins in HRMC treated by miR‐25 mimic or inhibitor, miR‐93 mimic or inhibitor, and miR‐106b mimic or inhibitor; *, ** and *** indicate *P* <.05, *P* <.01 and *P* < .001, respectively. Data are shown as the mean ±SEM

The main clinical features of IgAN were abnormal deposition of IgA1 in mesangium, which caused the proliferation of mesangial cells and leaded to renal insufficiency. To further determine the effect of miR‐25/93/106b cluster on glomeruli, human renal mesangial cells (HRMC) were transfected with miR‐25/93/106b inhibitor or mimic. Cell proliferation and fibrosis levels were then measured. Our results showed that inhibition of miR‐25/93/106b increased the HRMC proliferation (Figure 5A). Inhibition of miR‐25/93/106b also increased mRNA and protein expression levels of *collagen 1a (I), collagen 4a (IV), Fibronectin and Acta2* which was analysed by qPCR and WB in HRMC (Figure 5B‐E). Interestingly, upregulation of TFRC was also detected in the HRMC treated by miR‐25 cluster inhibitor (Figure 5F, G).

### 3.6 MiR‐93/106b regulates HRMC proliferation and fibrosis by targeting CCND1

**FIGURE 6:**
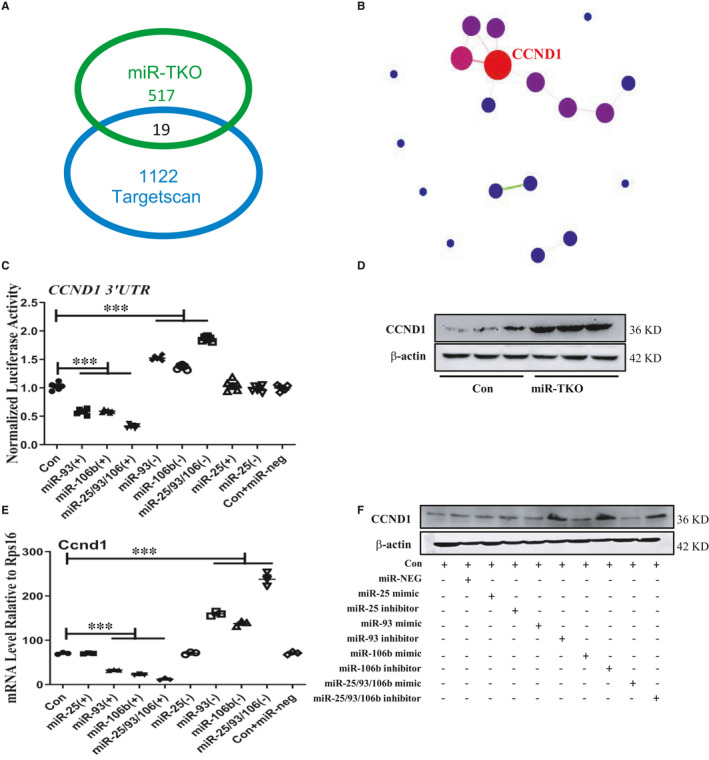
miR‐93/106b targets Ccnd1 in HRMC. A, The number of genes in the kidney with significant changes in 5‐mo‐old miR‐TKO mice and those identified as potential miR‐25/93/106b targets using the Targetscan database; B, PPI analysis from differential gene between miR‐TKO mice and miR‐93/106b targeting genes; C, Luciferase activity in HEK 293A cells that were transfected with the indicated 3’‐UTR reporter constructs showing binding of miR‐93/106b with the 3’‐UTR of *Ccnd1*; D, Western blot analysis for CCND1 from 5‐mo‐old miR‐TKO and wild‐type mice; E and F, quantitative real‐time PCR and western blot analysis for Ccnd1 from overexpression or inhibition of miR‐25, miR‐93 and miR‐106b treated HRMC. *, ** and *** indicate *P* < .05, *P* < .01 and *P* < .001, respectively. Data are shown as the mean ± SEM

Through the RNA‐seq results, 536 genes were significantly altered, and 19 of these genes were miR‐25/93/106b cluster potential targets analysed by crossing this cluster target database (Figure 6A). Through the analysis of overlapped 19 genes by bioinformatic protein network interaction, it showed the weight of Ccnd1, a potential target, was the most significant (Figure 6B). Luciferase assay showed that overexpression of miR‐93 and miR‐106b significantly inhibited Ccnd1 3’‐UTR luciferase activity, especially overexpression of total miR‐25 cluster (Figure 6C). The upregulated protein expression level of Ccnd1 was also detected in 5‐month‐old miR‐TKO mice as demonstrated by Western blot (Figure 6D). We also found that the protein and mRNA expression levels of Ccnd1 were increased in HRMC with miR‐93 and miR‐106b inhibitor compared with scrambled miRNA and miR‐25 inhibitor transfection (Figure 6E and F). These data identified both miR‐93 and miR‐106b could bind to the 3’‐UTR of its putative target Ccnd1.

**FIGURE 7:**
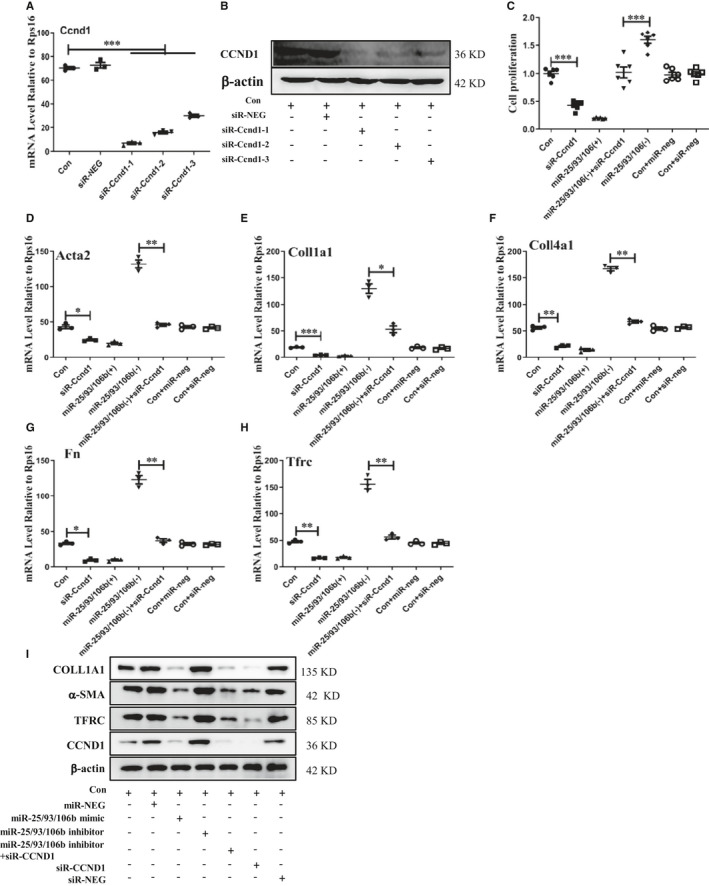
miR‐93/106b regulate cell proliferation and fibrosis in HRMC by targeting Ccnd1. A and B, Quantitative real‐time PCR and western blot analysis for Ccnd1 from HRMC treated by Ccnd1 siRNA; C, CCK8 analysis of cell proliferation level from treated HRMC by Ccnd1 siRNA, miR‐25/93/106b mimic or inhibitor, miR‐25/93/106b inhibitor plus Ccnd1 siRNA, and miRNA or siRNA negative control; D‐H, quantification of real‐time PCR analysis of *Coll4a1*, *Coll1a1, Fn*, Acta2 and *Tfrc* from treated HRMC by Ccnd1 siRNA, miR‐25/93/106b mimic or inhibitor, miR‐25/93/106b inhibitor plus Ccnd1 siRNA, and miRNA or siRNA negative control; I, Western blot analysis for COLL1A1, α‐SMA, TFRC and CCND1 from treated HRMC by Ccnd1 siRNA, miR‐25/93/106b mimic or inhibitor, miR‐25/93/106b inhibitor plus Ccnd1 siRNA, and miRNA or siRNA negative control. *, ** and *** indicate *P* < .05, *P* < .01 and *P* < .001, respectively. Data are shown as the mean ± SEM

To explore the mechanism of Ccnd1 underlying the involvement of miR‐25/93/106b in HRMC proliferation and fibrosis, we successfully decreased protein and mRNA level of Ccnd1 in HRMC cells with siR‐Ccnd1 (Figure 7A, B). We explore whether decreased protein level of Ccnd1 suppresses cell proliferation and fibrosis of HRMC with transfecting miR‐25/93/106b inhibitor. We found that inhibition of Ccnd1 could suppress miR‐25/93/106b inhibitor‐induced cell proliferation by CCK8 measurement (Figure 7C). Meanwhile, we also found that inhibition of Ccnd1 could decrease mRNA and protein expression level of fibrosis‐related gene (Coll4a1, Coll1a1, Fn and Acta2) and Tfrc in HRMC treated by miR‐25/93/106b inhibitor (Figure 7D‐I).

## 4 DISCUSSION

In the present study, we showed increased IgA/G and C3 deposition in glomeruli mesangium, which is consistent with serum IgA. We further illuminated the effect of miR‐25/93/106b cluster deficiency on the kidney, and showed their target Ccnd1 which could regulate mesangial cell proliferation and fibrosis.

Up to date, pathogenesis and treatment of IgAN are still an enigma because no exact animal model mimics the clinical IgAN features. The well‐known IgAN animal model is DdY mice.^27^ DdY mice also showed that IgA, IgG and IgM deposition was increased in the mesangial region of glomeruli, and there are upregulated IgA, IgG and IgM contents in the sera.^27^ Studies have shown that TFRC,^28^ as the IgA receptor on mesangial cells, significantly increased the expression level in IgAN. Our results also showed that TFRC deposition was significantly increased in the glomerular mesangial region, and miR‐93/106b inhibitor also increased the protein expression level of TFRC in vitro. Our results indicate that renal fibrosis and RAS‐related genes (*Fn, a‐SMA, Col1a1 and Col4a1*) are increased at mRNA and protein expression levels in 5‐month‐old miR‐TKO mice.

At present, the recognized pathogenesis of IgAN is the four‐hit theory,^29^ but the clinical detection is mainly kidney biopsy to detect the deposition of immune complex in the renal mesangial region, that is, the fourth strike, the deposition of immune complex in the mesangial region, causing the excessive proliferation of mesangial cells and the generation of fibrosis. Therefore, we mainly explored the effects of miR‐25 cluster on the proliferation and fibrosis of human renal mesangial cells. CCK8 detection results showed that the inhibition of miR‐25 cluster could promote the proliferation and fibrosis of mesangial cells in vitro. MiR‐25 cluster also regulates cell proliferation in different diseases.^30^, ^31^, ^32^, ^33^, ^34^ The results of luciferase assay showed that miR‐25/93/106b could negatively regulate the expression of Ccnd1 in mesangial cells, and the results of qPCR and WB in vitro and in vivo also indicated that the inhibition of miR‐93/106b could promote the expression of Ccnd1. It is well known that Ccnd1 is a key gene that regulates the stage of the cell cycle, and activation of its expression can promote cell proliferation. Next, we explored the role of Ccnd1 by inhibiting the expression of Ccnd1 in the inhibitor group of miR‐93 cluster. Inhibition of Ccnd1 expression could inhibit cell proliferation and cell fibrosis.

In summary, deficient of miR‐25 cluster induced IgAN‐like kidney disease, which illustrated miR‐25 cluster possibly played important role in the pathogenesis of IgAN, and our study also provides a new target for the treatment of IgAN in the future.

## CONFLICT OF INTEREST

All the authors declared no competing interests.

## AUTHOR CONTRIBUTION

**Hongchuang Ma:** Data curation (equal); Formal analysis (equal); Methodology (equal); Project administration (equal). **Xiang Li:** Formal analysis (equal); Methodology (equal). **Shanshan Yu:** Data curation (equal); Methodology (equal). **Yanling Hu:** Data curation (equal); Methodology (equal). **Meixiang Yin:** Formal analysis (equal); Methodology (equal). **Fubin Zhu:** Methodology (equal). **Licheng Xu:** Methodology (equal). **Tianhe Wang:** Methodology (equal). **Huiyan Wang:** Conceptualization (equal). **Hongzhi Li:** Conceptualization (equal). **Binghai Zhao:** Conceptualization (equal). **Yadong Huang:** Conceptualization (equal); Data curation (equal); Formal analysis (equal); Funding acquisition (equal); Investigation (equal); Project administration (equal).

## Supporting information

Supplementary Material
