# Supplementary material for: Deletion of the miR‐25/93/106b cluster induces glomerular deposition of immune complexes and renal fibrosis in mice
Source: J Cell Mol Med. 2021 Jul 1;25(16):7922–34. doi: 10.1111/jcmm.16721 (PMC8358857; doi:10.1111/jcmm.16721)
Supplement: Supplementary file 1 — Supplementary Material [file JCMM-25-7922-s001.docx]

Table S1 Ccnd1 3’UTR primers

| gene | Forward primer | Reverse primer |
| --- | --- | --- |
| Hsa-Ccnd1 | AATCTCGAGGCCCCGCGGAAGCAGCCTAGCACAGAC | AATGCGGCCGCGCCCTGCTGCAGTCCTTAACAGGCAGGGAT |

underlined sequences indicate the endonuclease restriction site

Table S2 mouse mRNA primers

| Gene | Forward primer | Reverse primer |
| --- | --- | --- |
| Fn | GAGTGGAAGTGTGAGCGACAT | TGAGTCTGCGGTTGGTAAATAG |
| Pai1 | GCCAGGGTTGCACTAAACAT | GCCTCCTCATCCTGCCTAA |
| Colla1 | CGTATCACCAAACTCAGAAGATG | ACCAGGAGGACCAGGAAGTC |
| Ccnd1 | TCTTCCATCACGGAGAGGTC | GATGCACTGCCTATGAGCAC |
| Acta2 | GTTCAGTGGTGCCTCTGTCA | ACTGGGACGACATGGAAAAG |
| Timp1 | AGGTGGTCTCGTTGATTTCT | GTAAGGCCTGTAGCTGTGCC |
| Col4a1  Tfrc  At1  At1a  At1b | ATCCGGCCCTTCATTAGC  GTTTCTGCCAGCCCCTTATTAT  AGGGTCTGGGCCATAGAACT  AACAGCTTGGTGGTGATCGTC  GGAAACAGCTTGGTGGTGAT | ACTGCGGAATCTGAATGGTC  GCAAGGAAAGGATATGCAGCA  CCACCACGCTCTTCTGTCTAC  CATAGCGGTATAGACAGCCCA  GTAAGATCGCTTCTGCCAGC |

Table S3 human mRNA primers

| Gene | Forward primer | Reverse primer |
| --- | --- | --- |
| Coll1a1 | GAGGGCCAAGACGAAGACATC | CAGATCACGTCATCGCACAAC |
| Coll4a1 | CAAGAGGATTTCCAGGTCCA | TCATTGCCTTGCACGTAGAG |
| Fn | GATAAATCAACAGTGGGAGC | CCCAGATCATGGAGTCTTTA |
| Acta2 | CTGAAGAGCATCCCACCCTG | CATCTCCAGAGTCCAGCACG |
| Ccnd1  Tfrc | AGAGGATGATTGCCGCCGT  GGCTACTTGGGCTATTGTAAAGG | CAACCACCCTGGTCTTGGATC  CAGTTTCTCCGACAACTTTCTCT |
